# Different types of exercise and myocardial angiogenesis regulation: A scoping review of cardiac‐specific evidence in animal models

**DOI:** 10.14814/phy2.70775

**Published:** 2026-03-04

**Authors:** Putri Karisa, Nova Sylviana, Aziiz Mardanarian Rosdianto, Hanna Goenawan, Mas Rizky Anggun Adipurna Syamsunarno, Setiawan

**Affiliations:** ^1^ Doctoral Program in Medical Science, Faculty of Medicine Universitas Padjadjaran Bandung Indonesia; ^2^ Cardiovascular and Metabolic Medicine Research Group, Faculty of Medicine Universitas Padjadjaran Bandung West Java Indonesia; ^3^ Department of Biomedical Sciences, Faculty of Medicine Universitas Padjadjaran Bandung Indonesia; ^4^ Physiology Molecular Laboratory, Biology Activity Division, Central Laboratory Universitas Padjadjaran Sumedang West Java Indonesia; ^5^ Center of Sport Science, Wellness, and Longevity, Graduate School Universitas Padjadjaran Bandung West Java Indonesia

**Keywords:** angiogenesis, cardiovascular, exercise, exercise physiology

## Abstract

Exercise promotes myocardial angiogenesis, yet molecular responses vary by modality, and cardiac‐specific evidence in healthy models remains fragmented. This study aimed to map biomolecular regulators of myocardial/coronary angiogenesis across exercise types in healthy animal models. PRISMA‐ScR scoping review. Literature searches were conducted in PubMed, Scopus, and SpringerLink. Eligible studies were structured exercise interventions in physiologically healthy animals reporting myocardial/coronary tissue angiogenesis biomarkers and/or structural microvascular indices; disease models and studies reporting only circulating markers were excluded. FITT parameters and outcomes were charted and synthesized by exercise type. From 4674 records, 23 studies were included across swimming, continuous treadmill/running, interval/HIIT, resistance, and combined training. Aerobic protocols most consistently upregulated the VEGF‐VEGFR axis with downstream PI3K/Akt‐eNOS/NO and context‐dependent HIF‐1α signaling. Interval/HIIT showed prominent endothelial activation (e.g., miR‐126 and CD34/KDR) alongside increased vascular indices. Resistance/combined training was less represented, but implicated AMPK‐PGC‐1α/FNDC5‐irisin signaling, VEGFR2/FLK‐1 upregulation, and eNOS/NO support in combined protocols. Exercise modality appears to recruit distinct upstream regulators that converge on myocardial angiogenic remodeling; evidence is dominated by male‐only models, and mechanistic data for resistance/combined training remain limited.

## 1 INTRODUCTION

Aligned with global priorities to reduce premature mortality from noncommunicable diseases through prevention and health promotion, sedentary behavior is a major contributor to the worsening of noncommunicable diseases. It reportedly increases the risk of CVD by 34% and is closely related to all‐cause mortality (Liang et al., 2022). Replacing sedentary behavior with physical activity or exercise is an effective strategy to improve quality of life and reduce the risk of CVD (Aune et al., 2021). Increased physical activity, including exercise, can reduce the risk of obesity, hypertension, and elevated glucose and cholesterol levels, thereby helping prevent CVD and its complications (Barone Gibbs et al., 2021).

Exercise plays an important role in promoting various physiological adaptations in all organs, particularly the heart. It reportedly improves and maintains cardiorespiratory fitness in healthy, at‐risk, and CVD patients (Imboden et al., 2019; Kaminsky et al., 2019). Physical exercise alters blood flow, muscle contraction, and oxygen levels, which are associated with mechanical hemodynamics in the vasculature. These events are key signals that induce vascular remodeling and activate growth factors associated with endothelial cell proliferation, migration, and lumen formation in the muscle (Kivelä et al., 2023). Increased blood flow velocity leads to stress changes that trigger blood vessel enlargement and capillary formation through a process called angiogenesis (Arany et al., 2005; Kivelä et al., 2023).

Angiogenesis is a key physiological process that enhances oxygen and nutrient delivery to active muscles, and increased capillarity can promote cardiovascular endurance and health (Liu et al., 2022). Angiogenesis starts with the release of pro‐angiogenic signals by the myocardium. Hypoxic conditions are believed to be the strongest factor inducing increased expression of pro‐angiogenic genes that stimulate blood vessel growth to fulfill the need for adequate oxygen and nutrient supply to maintain cardiac mass and function (Warmke et al., 2018).

Studies have reported various molecular mechanisms by which exercise can improve cardiovascular health, including adaptive changes in cardiac function and structure (Ashor et al., 2015; Gunadi et al., 2019; Lerchenmüller et al., 2022; Shibata & Levine, 2012), metabolism (Lu et al., 2015; Wilson & Johnson, 2000), signaling pathways (Bei et al., 2017; Kim et al., 2008; Xi et al., 2016), and vascular remodeling (Hieda et al., 2021; Xi et al., 2016, 2021). Angiogenesis induced by physical exercise is related to many angiogenic factors, including vascular endothelial growth factor (VEGF), a potent pro‐angiogenic factor in vasculogenesis and angiogenesis that induces cell proliferation, inhibits apoptosis, and increases vascular permeability, vasodilation, and recruitment of inflammatory cells to the site of injury (Bekhite et al., 2011; Iemitsu et al., 2006; Koch & Claesson‐Welsh, 2012). Exercise presumably impacts cardiac angiogenesis by increasing VEGF expression through various mechanisms, including hypoxia‐induced factor 1 alpha (HIF‐1α), peroxisome proliferator‐activated receptor gamma coactivator 1 alpha (PGC‐1α), follistatin‐like protein 1 (FSTL‐1), and others (Arany et al., 2005; Sylviana et al., 2018, 2022; Xi et al., 2021). However, there is conflicting evidence that exercise does not have a long‐term effect on the growth of new blood vessels in the heart (Esfahanni et al., 2014; Marini et al., 2008).

The inconsistent findings on exercise‐induced expression of angiogenesis‐related markers likely reflect heterogeneity in the exercise stimulus itself, which is best describe using the FITT (frequency, intensity, time, and type) framework (Bellafiore et al., 2019; Sylviana et al., 2022; Yeo & Lim, 2022). In terms of type, exercise categorized based on the oxygen consumption that occurs during the training (Knuttgen, 2007). Different training modalities can improve cardiovascular health through partially distincy physiological pathways (Guo & Wang, 2025). Endurance/aerobic exercise consistently enhances endothelial function (often quantified by flow‐mediated dilation) and improves maximal cardiac output via structural and functional cardiac adaptations that increase stroke volume and central blood volume (Smith et al., 2014). Resistance training (RT) is increasingly recognized as a safe and effective modality for improving cardiovascular health and traditional risk factors (e.g., blood pressure, glycemic control, lipids, and body composition), while its effects on arterial stiffness may vary by population and program characteristics, highlighting the need to extract and report FITT in detail (Cornelissen & Smart, 2013). Finally, combined training (CT) may provide additive or synergistic benefits by integrating aerobic‐driven vascular adaptations with strength‐driven improvements in muscular fitness and metabolic regulation, contributing to favorable changes in blood pressure and cardiometabolic markers (Schroeder et al., 2019).

Detailed knowledge of the molecular pathways governing exercise‐induced angiogenic remodeling may help consolidate mechanistic hypotheses and guide biomarker‐oriented research on cardiovascular adaptation (Bernardo et al., 2018). Variability in observed responses to angiogenesis markers is well recognized in the coronary literature. It has long been attributed to heterogeneity in training characteristics (magnitude, type, and intensity), age, and experimental context, underscoring the importance of interpreting molecular signals through the exercise stimulus rather than labels alone (An et al., 2024; Damay et al., 2023; Sugiono et al., 2024). Although several recent syntheses have compared exercise effects, many emphasize dose dimensions (e.g., intensity and duration) or summarize vascular/angiogenesis outcomes using circulating or peripheral markers, which may not directly reflect myocardial remodeling. Although prior reviews have substantially advanced the field of exercise‐cardiac remodeling, they often discuss angiogenesis as one component within broader remodeling hallmarks or signaling networks, and summarize vascular adaptations at a systemic level rather than explicitly consolidating myocardium‐specific angiogenesis regulators across multiple exercise modalities and training prescriptions (Bloor, 2005; Qiu et al., 2022; Schüttler et al., 2019). In parallel, recent comparative evidence syntheses have contrasted aerobic exercise (AE) with resistance training (RT), primarily using functional and body‐composition endpoints in aging populations, leaving unresolved how different exercise modalities relate to the molecular regulation of cardiac adaptation, including angiogenesis (Khalafi et al., 2025). Accordingly, this review aims to systematically map the biomolecular regulators implicated in exercise‐related myocardial angiogenesis across diverse exercise modalities, and to summarize how variation in training prescriptions and study context may relate to heterogeneity in reported molecular signature, thereby clarifying what is currently supported by the literature and delineating priorities for future mechanistic studies.

## 2 METHODOLOGY

### 2.1 Ethical consideration

The articles included in the reviewed study were from previously published journals and were accessible to the reviewers. It means that no specific ethical review was required.

### 2.2 Study design and review question

This study used the Preferred Reporting Items for Systematic Reviews and Meta‐Analyses for Scoping Reviews (PRISMA‐ScR) framework (Tricco et al., 2018). A PRISMA‐ScR checklist is provided as Table S1. The design was used to investigate various types of exercise that can affect cardiac angiogenesis. Therefore, the review question is: How do different types of exercise affect regulators of cardiac angiogenesis in biomolecular pathways?

### 2.3 Search strategy

Electronic databases, including PubMed, Scopus, and SpringerLink, were used to obtain primary studies in any language. In the article search, three general concepts were used to identify medical subject headings (MeSH) using keywords: exercise, angiogenesis, and cardiovascular. All keywords were searched for in the title, abstract, and field. Boolean operators were used in the search. For keywords and subjects identified in the same concept, “OR” was used, while “AND” was used to link key concepts. Data retrieval from the database was conducted from September to November 2025. Exercise intervention was the independent variable, and cardiac angiogenesis was the dependent variable, allowing exploration of different types of exercise that could improve angiogenesis in the healthy heart. The search query is reported in Table S2.

### 2.4 Study selections

All search records were downloaded from three databases (PubMed, Scopus, and SpringerLink). The search results were checked for duplication using the Rayyan software. Three reviewers (PK, NS, and MRAAS) independently screened the search results based on identified titles and abstracts. After obtaining the full‐text versions of all identified studies, one of the reviewers collected the relevant full text. The three reviewers also independently assessed the full text for inclusion using predefined criteria. When there was a discrepancy between the two reviewers' assessments, the other reviewer (SS) was included to resolve it.

### 2.5 Inclusion and exclusion criteria

Inclusion criteria were determined according to the PCC (Population, Context, and Concept) methodology. Eligibility criteria were defined using the PCC framework recommended for scoping reviews.

1. Type of participants: original experimental studies involving physiologically healthy animal models (any age/sex; irrespective of baseline training status). Studies using pathological animal models, including but not limited to coronary artery disease, myocardial infarction, heart failure, diabetes, hypertension, obesity‐related cardiometabolic disease, or other induced/clinical disease states. During screening we considered human exercise studies potentially eligible only if they reported myocardial/coronary‐specific angiogenesis outcomes derived from cardiac or coronary‐source samples (e.g., myocardial tissue or coronary‐source specimens). However, no human studies meeting these cardiac‐specific outcome requirements were identified, and therefore no human studies were included and the final synthesis is restricted to preclinical animal evidence.
2. Context: Any structured exercise intervention with a prespecified protocol was eligible, including continuous endurance/aerobic training, interval‐**based** training (e.g., HIIT or sprint‐interval protocols), resistance/strength training, and concurrent/combined training (CT), as well as other structured programs where FITT parameters could be charted. No minimum requirements were imposed for frequency, intensity, or duration at the eligibility stage; instead, FITT parameters were extracted during data charting to contextualize the training stimulus.
3. Concept: Studies were included only if they reported myocardium‐ or coronary‐specific angiogenesis outcomes, defined as at least one of the following:

- Cardiac tissue biomarkers related to angiogenesis (e.g., mRNA/protein expression of VEGF/VEGFR, HIF‐1α, eNOS/NO‐related pathways, PGC‐1α, FSTL1, angiopoietins, FGF‐related markers, miRNA regulators, and other angiogenic mediators) measured in myocardial/coronary tissue; and/or
- Structural myocardial microvascular indices consistent with angiogenesis/vascular remodeling (e.g., capillary density, capillary‐to‐myocyte ratio, arteriolar density/diameter, and microvascular area), quantified using histology/histomorphometry, immunohistochemistry/immunofluorescence, or other validated tissue‐based techniques.
- Studies reporting only circulating (serum/plasma) angiogenesis‐related biomarkers without myocardial/coronary endpoints were not eligible because they are not cardiac‐specific and may reflect contributions from multiple tissues (e.g., skeletal muscle).

Studies were additionally excluded if they:

1. Did not use an experimental exercise‐training intervention with a prespecified protocol (e.g., observational physical activity studies, cross‐sectional comparisons, or acute exercise sessions without a structured training program);
2. Combined exercise with co‐interventions that could not be disentangled from the exercise effect (e.g., pharmacological agents, surgery, dietary manipulation, and hypoxic chambers), unless an exercise‐only comparator arm was available;
3. Were non‐original research (reviews, editorials, commentaries, and conference abstracts without full methods/results), except when used for background screening; or
4. Were not available in full text or lacked sufficient methodological/outcome detail to enable data charting.

### 2.6 Data extraction and analysis

Three reviewers (PK, NS, and MRAAS) independently extracted data from the study results using Excel, with high agreement among them (≥95%). Any disagreement was resolved by consensus with another reviewer (SS). One reviewer (PK) extracted data from all the included studies. The extracted data can be seen in Table 1 (Study Characteristics) and Table 2 (Findings Related to Angiogenesis). The extracted data includes: The extracted data can be seen in Table 1 (Study Characteristics) and Table 2 (Findings Related to Angiogenesis). The extracted data include: (1) first author's name and year of publication, (2) animal species, size, and sex, (3) analysis method, (4) intervention protocol (frequency, intensity, type, and duration of exercise), (5) results, and (6) conclusions or significance of the study. When the data was not explicitly reported, it was recorded as not available (N/A). The included studies will then be analyzed and mapped based on the type of exercise and the molecular pathway that induces angiogenesis based on the findings of gene expression and structural changes assessed by histological examination.

**TABLE 1:** Study and intervention characteristics.

Study (author, year)	Characteristics sample	Analysis/ specimens	FITT concept (frequency, intensity, type, and time)
Iemitsu et al. (2006)	24 male Wistar rats	PCR, IHC/Cardiac tissue	F: 5 days/week, 7 weeks I: N/A T: Swimming T: 15 min (2 days) and increase until 90 min/day
Marini et al. (2008)	24 male albino Sprague–Dawley rats	RT‐PCR, immunohistochemistry/cardiac tissue	F: 3 days/week, 5 weeks I: Moderate intensity (60% VO2 max) T: Continuous training (Treadmill) T: 60 min/day
Da Silva Jr et al. (2012)	21 Female Wistar rats	RT PCR, Western blot, H&E/cardiac tissue	F: 5 days/week, 10 weeks. However, group T2 trained twice daily in week 9 and three times daily in week 10. I: Swimming with a load of 5% of body weight on the rat's tail T: Swimming T: 60 min/day
Hassan and Kamal (2013)	40 male Albino rats	ELISA, H&E/cardiac tissue	F: 5 days/week, 6 weeks I: Progressive low to moderate (Week‐2: 50% BW, Week‐3 and 4: 60% BW, and Week‐5 and 6: 70% BW) T: Swimming T: 30 min/day
Esfahanni et al. (2014)	20 male Wistar rats	ELISA/coronary blood	F: 3 days/week, 4 weeks I: 30–100% of BW (weight increased over 2 weeks) T: Resistance training T: 18 repetition
Xiao et al. (2016)	10 male C57BL/6 mice	qRT‐PCR, immunofluorescence/cardiac tissue	F: Twice/day, 4 weeks I: N/A T: Swimming T: 90 min/day
Ghorbanzadeh et al. (2017)	40 male Wistar rats	ELISA, RT‐PCR, immunohistochemical/cardiac tissue	F: Every day, 8 weeks I: N/A (Running distance >2000 m per day) T: Continuous training (Treadmill) T: N/A
Ardakanizade (2018)	18 male Wistar rats	RT‐PCR/left ventricle tissue	F: 5 times/week, 10 weeks I: N/A T: Swimming T: 60 min (MT) and 270 min (LT) per day
Sylviana et al. (2018)	14 male BALB/c mice	Semiquantitative‐PCR, H&E/heart tissue	F: N/A I: N/A T: Swimming T: N/A
Bellafiore et al. (2019)	63 male Swiss mice	Western blot, immunostaining/left ventricle tissue	F: 5 days/week I: low (T15), moderate (T30), and high (T45) T: Treadmill (Endurance training) T: 150 min (T15), 225 min (T30), and 300 min (T45)
Verboven et al. (2019)	26 male Sprague–Dawley rats	Fibrosis (Chondrex), capillary density (immunohistochemical), Western blot/cardiac tissue	F: 5 days/week I: 18 m/min (MIT) & 10 sessions 18 m/min, separated by 1 min active rest (HIIT) T: Continuous training (Treadmill) and HIIT T: 60 min/ day
Pourheydar et al. (2020)	21 male Wistar rats	ELISA, IHC and HE/left ventricle tissue	F: 7 weeks/ every day I: Mild exercise 17 m/min T: Continuous training (Treadmill) T: 10 min (first day), and increase every day (+5 min) until 30 min.
Sabzevari Rad et al. (2020)	40 male Wistar Rats	RT‐PCR, ELISA/cardiac tissue	F: 6 days/week, 6 weeks I: High intensity (95–100% VO_2_ max) T: HIIT T: 30 min/day
Chen et al. (2021)	40 Zebra fish	H&E, qPCR, Western blot/cardiac tissue	F: 6 days/week, 4 weeks I: 23.5 cm.s T: Swimming T: 240 min/day
Akbari et al. (2022)	30 male Wistar rats	RT‐PCR/left ventricle tissue	F: Every day, 6 weeks I: 22 m/min for 30 min (CT) and 39 m/min for 5 × 2 min with interval 5 × 1 min intervals at 12 m/s (IT) T: Continuous training (Treadmill) and HIIT T: 30 min/day
Rezaei et al. (2022)	49 female old Wistar rats	RT‐PCR/cardiac tissue	F: 3 sessions/ week, 8 weeks I: 10 sets of 1 min Moderate (14 m/min – 28 m/min; 50% VO_2_ max) and high intensity (20 m/min – 34 m/min; 70% VO2 max) with each week increasing by 2 m/min and 2 min of rest between sets T: Moderate and high intensity interval training (HIIT) T: 30 min/day
Soori et al. (2022)	24 male Wistar rats	qRT‐PCR, ELISA, Mason's trichome staining/cardiac tissue	F: 5 sessions/week, 6 weeks I: High intensity: progressive 80%, 90%, and 100% maximal speed (HIIT) and moderate intensity: 65% and 70% maximal speed continuous training (Treadmill) T: High‐intensity interval training (HIIT) and continuous training (Treadmill) T: 10–16 min with each interval separated by 2 min (HIIT) and 15–25 min (weeks‐3) (CET)
Sylviana et al. (2022)	20 male Wistar rats	Western blot/left ventricle tissue	F: 5 days/week, 12 weeks I: 10 m/min (LI), 20 m/min (MI), and 30 m/min (HI) T: Continuous training (Treadmill) T: 30 min/day
Yeo and Lim (2022)	24 male Sprague–Dawley rats	Western blot, immunohistochemistry/left ventricle muscle	F: 8 weeks I: Aerobic (6 m/min for 30 min, increased 13 m/min (60% VO_2_ max for 60 min)), resistance training (50%–130% BW), and combined training (AE: 13 m/min for 30 min and RT just 6 repetitions) T: Aerobic (Treadmill), Resistance training, and Combined training: T: 60 menit, 12 repetition for RT
Farhadi et al. (2024)	30 male Wistar rats	Western blot, H&E/cardiac tissue	F: 5 sessions/week, 8 weeks I: 10–20 m/min (first week), 22–26 m/min (2–8 weeks) T: Continuous training (Treadmill) T: N/A
Karisa, Sylviana, Goenawan, et al. (2025)	20 male Wistar rats	qRT‐PCR/cardiac tissue	F: 5 days/week, 15 days (AE) and 8 weeks (CE) I: Moderate intensity – 20 m/min T: Continuous training (Treadmill) T: 30 min/day
Ma et al. (2025)	18 female Sprague–Dawley rats	H&E, IHC, qRT‐PCR, Western blot/cardiac tissue	F: 5 days/week, 8 weeks I: N/A T: Swimming with a weight equivalent to 3% of body weight on the tail T: 60 min/day
Olamazadeh et al. (2025)	21 male NMRI mice	ELISA, RT‐PCR, Western blot, and immunohistochemistry (IHC)/cardiac tissue	F: 3 sessions/week, 8 weeks I: Progressive load (started from 30% of BW, and the load was increased up to twice of their BW) T: Resistance training (RT) T: 20 repetitions

**TABLE 2:** Angiogenesis‐Related Findings.

Study	Type exercise	Result	Importance
Iemitsu et al. (2006)	Swimming	There was an increase in total capillary density in the heart and the ratio of capillaries to myocytes (*p* < 0.05) VEGF mRNA (*p* < 0.05) and protein (*p* < 0.01) were increased There was an increase in Flt‐1 and Flk‐1 mRNA and protein (*p* < 0.05) There was an increase in p‐Akt and eNOS & p‐eNOS expression (*p* < 0.01), but no difference was found in Akt protein	Exercise appears to enhance cardiac angiogenesis by upregulating the VEGF axis, as evidenced by higher VEGF expression, increased Flt‐1 and Flk‐1, and activation of downstream PI3K/Akt signaling, as reflected by increased phosphorylated Akt. This signaling promotes nitric oxide bioavailability by increasing eNOS and phosphorylated eNOS levels, supporting endothelial function and capillary growth. Consistent with this mechanism, total capillary density and the capillary‐to‐myocyte ratio are increased compared with sedentary controls.
Marini et al. (2008)	Continuous Training (Treadmill)	There was an increase in the number and density of capillaries and Vwf(+) (*p* < 0.05); detraining decreased this increase, but the number remained higher than in the control. New sprout growth was found in the exercise group There was an increase in VEGF, KDR, and HIF‐1α expression (*p* < 0.05). However, it decreased in the detrained group Although increased, FGF levels in the exercise group were not significantly different from those in the control group	Exercise likely promotes cardiac angiogenesis through a hypoxia‐mediated HIF‐1α pathway that increases VEGF and its receptor KDR, driving endothelial sprouting and higher capillary number and density with greater vWF positivity. With detraining, HIF‐1α, VEGF, and KDR signaling decline, and vascular indices fall but remain above control levels, indicating partial retention of the exercise‐induced microvascular adaptation. At the same time, FGF appears to play a minor role.
Da Silva Jr et al. (2012)	Swimming (T1 = continuous load, T2 = progressive load)	There was an increase in the capillary‐myocardial fiber ratio (*p* < 0.0001) compared to the control group VEGF and miRNA‐126 increased in T1 (*p* < 0.05) and T2 (*p* < 0.001) Signal targets: Raf‐1, PI3K, Akt1, eNOS increased in the T1 < T2 group ERK1/2 increased in T1 = T2. However, this was accompanied by a decrease in Spred‐1 and PI3KR2 in T1 and T2	Exercise increased miRNA‐126 expression through VEGF and increased angiogenic pathways (MAPK and PI3K/Akt/eNOS). Exercise decreased Spred‐1 protein expression, which interferes with angiogenesis and cell migration, thereby supporting MAPK, Raf‐1, and ERK1/2 phosphorylation signaling pathways.
Hassan and Kamal (2013)	Swimming	VEGF and cardiac glycogen levels were found to be significantly increased (*p* < 0.001) No difference in Caspase‐3 levels Histologically, a characteristic feature in the ET group was increased capillary density (angiogenesis) and cardiomyocyte hypertrophy	Increased capillary density (angiogenesis) was significantly higher in the exercise group, correlating with serum VEGF levels. Steroid administration in the exercise group also increased VEGF levels, but the angiogenic response was only mild.
Esfahanni et al. (2014)	Resistance Training	Serum NO, VEGF, and Flt‐1 increased in the exercise group, but there was no significant difference compared with the control group.	RT can induce angiogenic factors, but it requires a training duration of >8 weeks for maximum results. A short training period leads to increased VEGF expression in endothelial cells, rendering the results indistinguishable from those in the control group.
Xiao et al. (2016)	Swimming	Increased cell size and number of cardiomyocytes in the exercise group (*p* < 0.05) Increased CD34 (*p* < 0.01) and CD34/PDGF (p < 0.05) in the exercise group, accompanied by a decrease in BNP (*p* < 0.05)	Compared with the control, the swimming protocol increased CD34 and CD34/PDGF, consistent with increased capillarization/angiogenesis and the involvement of the PDGF pathway in vessel stabilization. Increased cell size/ANP, along with decreased BNP, supports a pattern of physiological remodeling (adaptive hypertrophy) parallel to the angiogenic response.
Ghorbanzadeh et al. (2017)	Continuous Training (Running)	Significant increase in p‐Akt (*p* < 0.01) Significant increase in p‐ERK1/2 (*p* < 0.05) Significant increase in miRNA‐126 (*p* < 0.01) Significant increase in miRNA‐210 (*p* < 0.01) Immunohistochemical examination showed a significant increase in the number of CD31+ cells in the exercise group compared to the control group (*p* < 0.05)	Running exercise in this study induced hypoxia‐mediated angiogenesis, as evidenced by increased miR‐210 (hypoxamiR), reflecting the hypoxia response and supporting the neovascularization program. In parallel, increased miR‐126, known to regulate cardiac angiogenesis by activating Akt and ERK1/2, was consistent with increased p‐Akt/p‐ERK1/2 and immunohistochemical evidence of CD31+ cells (increased capillarization).
Ardakanizade (2018)	Swimming (LT = Long term & MT = Mild term)	VEGF‐B, MEF2C, and MMP‐2 were found to be higher in the LT group than in the MT and Control groups (*p* = 0.001). However, ANG‐1 and HDAC4 expression increased significantly in the MT group (p = 0.001) and HDAC4 decreased in the LT group (*p* = 0.001) Oxidative stress and NO were found to be higher in the LT group than in the MT and Control groups (*p* = 0.001) CK and LDH increased in both exercise groups compared to the control group (LT (*p* = 0.001) > MT (*p* = 0.025))	Long‐term swimming (LT) appears to shift the response towards shear/hypoxia‐redox signaling (increased oxidative stress and NO), which activates the angiogenesis‐remodeling program via MEF2C and VEGF‐B, accompanied by increased MMP‐2, which facilitates ECM degradation and sprouting/vascular remodeling. In contrast, mild‐term (MT) emphasizes the stabilization/maturation phase of vessels through increased ANG‐1 with HDAC4, whereas in LT, HDAC4 decreases, consistent with the derepression of adaptive genes in chronic exposure. Increased CK/LDH (LT>MT) indicates higher tissue load/damage in LT, which may reinforce the redox–NO stimulus and vascular remodeling.
Sylviana et al. (2018)	Swimming	H&E: hypertrophy was observed in cardiac muscle cells There was an increase in PGC‐1α expression and a decrease in HIF‐1α in the swimming group compared to the control group (*p* < 0.05)	Swimming triggers physiological hypertrophy of cardiac muscle cells and promotes oxidative/mitochondrial adaptation via PGC‐1α, thereby improving myocardial oxygen handling and supporting angiogenesis. The resulting increase in oxygen delivery and decrease in cellular hypoxia stress are consistent with reduced HIF‐1α expression, indicating a shift from acute hypoxia signaling to a long‐term remodeling program mediated by PGC‐1α, which can maintain capillarization alongside adaptive hypertrophy.
Bellafiore et al. (2019)	Treadmill (Endurance training) (T15 = 15 days, T30 = 30 days, T45 = 45 days)	VEGFR‐1/Flt‐1 increased (T15 > T30 > Control>T45, *p* < 0.05) VEGFR‐2/Flk‐1 increased (T45 > T15 > T30 > Control, *p* < 0.05) HIF‐1α increased significantly only in the T15 group iNOS increased significantly in T15 and increased sharply in T30 compared to control (*p* < 0.05)	VEGFR‐1/Flt‐1 and HIF‐1α expression increased with light‐intensity exercise, while VEGFR‐2/Flk‐1 levels increased progressively with increasing workload. Conversely, iNOS protein was modulated by moderate‐intensity exercise. HIF‐1α is a central regulator of the exercise‐induced angiogenesis pathway in the myocardium. HIF‐1α may act as an upstream regulator of VEGFR‐1 and iNOS in modulating cell proliferation and vascular relaxation.
Verboven et al. (2019)	HIIT	Number of blood vessels increased (HIIT > MIT > Control) (*p* < 0.05) Increased Endothelin‐1 (Control > HIIT > MIT) Increased NOX2 (MIT > Control > HIIT) % fibrosis (MIT > HIIT) (*p* < 0.05)	HIIT and MIT interventions have different beneficial effects on the heart. HIIT is superior to MIT in increasing blood capillary density, thereby optimizing myocardial circulation and metabolism.
Pourheydar et al. (2020)	Continuous Training (Treadmill)	There was an increase in VEGF, NF‐κB, and CD31 (*p* < 0.001) in the exercise group compared to the control group However, there was a decrease in TSP‐1 (*p* < 0.001)	Interestingly, VEGF in the elderly exercise group was higher than in the young non‐exercise group, accompanied by increased NF‐κB expression and decreased TSP‐1. These results are consistent with the angiogenesis assessment by CD31 immunohistochemistry, which shows that exercise increases angiogenesis compared with the non‐exercise group.
Sabzevari Rad et al. (2020)	HIIT	The expression of miR‐126, VEGF, and Raf‐1 increased in the HT group compared to the HC group (*p* < 0.05). However, Spred‐1 levels decreased in the HT group.	In healthy trained subjects, increased miR‐126 is consistent with suppression of Spred‐1, a negative regulator of the Ras/Raf pathway. Reduced Spred‐1 likely releases inhibitory control, allowing stronger VEGF signaling and greater downstream activation of Raf‐1 and the MAPK/ERK cascade. Together, the pattern of higher miR‐126, VEGF, Raf‐1, and lower Spred‐1 supports an exercise‐induced pro‐angiogenic program that promotes endothelial survival and proliferation and ultimately enhances myocardial capillarization compared with healthy controls.
Chen et al. (2021)	Swimming	Relative fluorescence of VEGF increased (*p* < 0.001) The myocardium area increased in the control group (*p* < 0.001) mRNA expression (IGF‐1, PI3K, Akt1, mTOR, rps6kb1b, CITED4a, PGC‐1α, MAPK, HIF‐1α, and VEGF) and protein (p‐PI3K and p‐Akt) increased	Aerobic swimming exercise in zebrafish enhances physiological hypertrophy, angiogenesis, mitochondrial fusion, and autophagy.
Akbari et al. (2022)	Continuous Training (CT) and Interval Training (IT)	miR‐126 was significantly increased in the IT group (*p* < 0.05) miR‐222 was significantly increased in both exercise groups compared to the control (IT> CT, *p* < 0.05) There was a significant increase in miR‐29A in the IT and CT groups compared to the control group (IT > CT, *p* < 0.05)	Interval training (IT) appears to trigger a stronger angiogenic response than continuous training (CT), characterized by a dominant increase in miR‐126 (highest in IT), reflecting endothelial activation and promotion of sprouting/capillarization. Increased miR‐222 and miR‐29a in IT and CT (IT > CT) indicate additional modulation of vascular remodeling and extracellular matrix (maturation/stabilization), suggesting that overall IT likely drives greater angiogenesis and remodeling than CT.
Rezaei et al. (2022)	Moderate & High Intensity Interval Training	There was an increase in CD34 and KDR expression in the exercise group (HIIT > MIT) compared to the control (*p* = 0.0001). However, CRP levels decreased in the exercise group.	Exercise appears to enhance endothelial angiogenic signaling, with higher CD34 and KDR (VEGFR2) levels indicating increased endothelial cell activation and capillary potential, and a greater effect in HIIT than in MIT. The concurrent reduction in CRP suggests that this pro‐angiogenic adaptation occurs alongside dampened systemic inflammation, which may further support vascular remodeling and endothelial function.
Soori et al. (2022)	High‐Intensity Interval Training (HIIT) and Continuous Exercise Training (CET, Treadmill)	There was an increase in VEGF (*p* < 0.05 for HIIT group compared to control and CET) and FGF‐2 (*p* < 0.05 for CET group compared to HIIT and control) %fibrosis was significantly lower in CET & HIIT group compared with control (*p* = 0.01)	HIIT and CET decreased myocardial fibrosis, but fibrosis was lower in the CET group than in the HIIT group. HIIT significantly increased VEGF expression, but there was no significant difference between the CET and control groups. Interestingly, FGF‐2 expression was higher in CET than in HIIT. Different types and intensities of exercise influence the mechanisms of hypertrophy, fibrosis, and angiogenesis adaptation.
Sylviana et al. (2022)	Continuous Training (Treadmill)	There was an increase in HIF‐1α (*p* < 0.05) (HI > MI > LI > Control), PGC‐1α (*p* < 0.01) (LI > MI > HI > Control), VEGF (*p* < 0.05) (HI, MI, LI, control) and CD34+ (*p* < 0.01) (HI > MI > LI > Control) in the exercise group	There was a significant positive correlation between HIF‐1α and VEGF in the LI and MI groups, but no correlation in the MI group. On the other hand, a significant correlation between PGC‐1α and VEGF was only observed in the MI group. These results indicate that, in LI and HI, angiogenesis is regulated by HIF‐1α, whereas in MI it is induced via the PGC‐1α pathway.
Yeo and Lim (2022)	Aerobic Exercise (AE) (Treadmill), Resistance Training (RT), and Combined Training (CT)	eNOS increased in AE and CT (*p* < 0.05). In RT, it increased but was not significant HIF‐1α only increased significantly in AE (*p* < 0.05) PGC‐1α increased in RT < AE < CT (*p* < 0.05) VEGF and Ang‐2 significantly increased in AE (*p* < 0.05), and increased nonsignificantly in RT and CT FLK‐1 significantly increased in RT and CT (*p* < 0.05) Immunohistochemistry results showed increased CD31 in AE and CE compared to RT	Aerobic exercise (AE) most consistently induces myocardial angiogenesis by activating NO‐eNOS and the PGC‐1α/HIF‐1α/VEGF pathway, accompanied by increased ANG‐1, which supports vascular stability/permeability in the aging heart. Resistance exercise (RT) may elicit a different response: a non‐significant increase in eNOS, potentially offset by increased endothelin‐1 (which can inhibit eNOS activation). In contrast, an increase in FLK‐1/VEGFR2 (in RT and CT) may act as a compensatory mechanism when VEGF does not increase significantly.
Farhadi et al. (2024)	Continuous training (Treadmill)	There was an increase in HIF‐1α, VEGF, and Tie‐1 in the exercise group (*p* < 0.01), and hypoxia comparable to the hypoxia‐induced group was found p‐Akt increased significantly only in the exercise group (*p* < 0.001) There was an increase in capillary density in the exercise group compared to the control group	Exercise appears to trigger a hypoxia‐like angiogenic program, with higher HIF‐1α comparable to hypoxia induction alongside higher VEGF and Tie‐1, indicating endothelial activation/remodeling. The exercise‐specific p‐Akt suggests enhanced VEGF/PI3K/Akt signaling supporting angiogenesis, consistent with increased capillary density.
Karisa, Sylviana, Goenawan, et al. (2025)	Continuous Training (Treadmill)	There was an increase in HIF‐1α in the acute exercise (*p* = 0.006) and chronic exercise groups compared to the control group (*p* = 0.004), but an increase in FIH and PHD expression in the exercise group accompanied this.	Acute and chronic exercise appear to activate hypoxia signaling, as reflected by higher HIF‐1α levels than in control. Concurrent increases in FIH and PHD suggest a compensatory negative feedback loop that limits HIF‐1α transcriptional activity and promotes its hydroxylation and degradation, thereby fine‐tuning the angiogenic response and preventing excessive or prolonged hypoxia‐driven signaling during training.
Ma et al. (2025)	Swimming	CD31 immunostaining was increased in swimming group (*p* < 0.05) TESC protein (*p* < 0.01) and its targets increased phosphorylation levels of C‐SRC and IGF1R downstream of TESC (*p* < 0.05) Angiogenesis‐related proteins and genes increased PI3K, p‐AKT/AKT, VEGFA, and VEGFR2 (*p* < 0.05)	Swimming exercise significantly increases physiological hypertrophy and angiogenesis in the heart myocardium. This study shows that TESC (Teskalsin) is a protein that promotes myocardial capillary formation by activating the C‐SRC/IGF1R pathway.PI3K/AKT/VEGFA signaling pathway. These results further support TESC's key regulatory role in exercise‐induced myocardial angiogenesis.
Olamazadeh et al. (2025)	Resistance Training (RT)	VEGF protein and mRNA significantly increased in the RT group (*p* < 0.05) Immunohistochemistry detection of VEGF protein was found to be significantly increased and higher in the RT group (*p* < 0.05) Irisin significantly increased in the RT > IR > Control group (*p* < 0.05) A strong correlation was found between irisin and VEGF in the RT group	Cardiac angiogenesis induced by RT is likely due to increased AMPK/PGC‐1α/FNDC5, which is cleaved to its active form, Irisin.

Abbreviations: AE, aerobic exercise; Ang‐1, angiopoietin‐1; Ang‐2, angiopoietin‐2; BW, body weight; CD34+, cluster of differentiation 34; CT, combine training; eNOS, endothelial nitric oxide synthase; FGF‐2, fibroblast growth factor 2; FLK1, fetal liver kinase‐1; FLT1, fms‐like tyrosine kinase‐1 atau VEGFR‐1; HIF‐1α, hypoxia‐inducible factor 1‐alpha; HIIT, high‐intensity interval training; MEF‐2C, myocyte enhancer factor 2c; miRNA, microRNA; MDA, malondialdehyde; NF‐kB, nuclear factor kappa‐light‐chain‐enhancer of activated B cells; P‐AKT, phosphorylated protein kinase B atau Akt; PGC‐1α, peroxisome proliferator‐activated receptor‐gamma 1‐alpha; RT, resistance training; TSP 1, thrombospondin‐1; VEGF, vascular endothelial growth factor; VEGFR‐1, vascular endothelial growth factor receptor‐ 1; VEGFR‐2, vascular endothelial growth factor reseptor‐2.

## 3 RESULTS

### 3.1 Study selection

**FIGURE 1:**
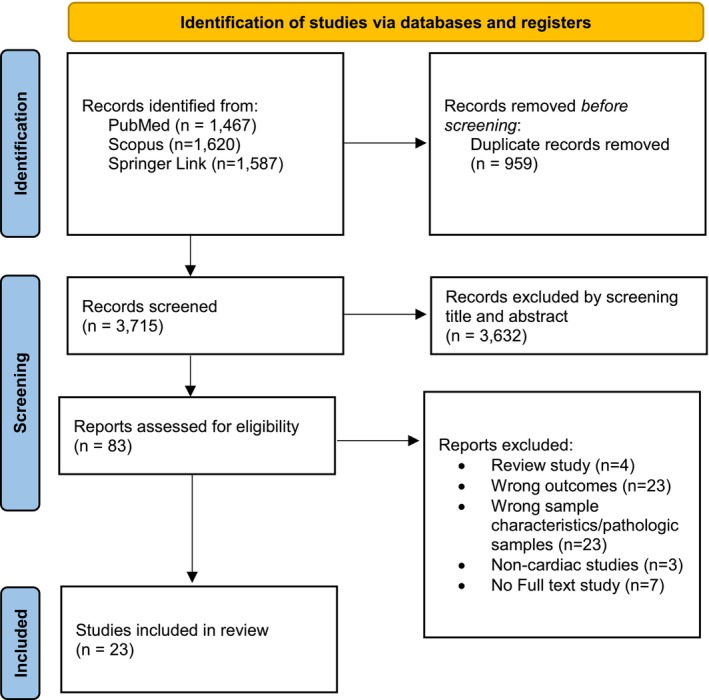
PRISMA flowchart of the study selection process. Adapted from Page et al. (2021).

The database search (PubMed, Scopus, and SpringerLink) retrieved 4674 records. All records were imported into Rayyan and 959 duplicates were removed. Two reviewers screened titles and abstracts and excluded 3632 records that did not meet the predefined eligibility criteria. The full texts of 83 articles were then assessed for eligibility, and reasons for exclusion were documented. Following full‐text review, 60 articles were excluded, leaving 23 studies for inclusion in the scoping review. The study selection process is presented in the PRISMA flow diagram (Figure 1). Importantly, no human studies met our cardiac‐specific outcome requirements (i.e., myocardial/coronary sample‐based angiogenesis outcomes). Therefore, all included studies were preclinical in vivo animal experiments.

### 3.2 Study and intervention characteristics

The included studies were published between 2006 and 2025 and comprised 23 preclinical in vivo experimental studies involving a total of 637 animals. Species/strains included Wistar rats (*n* = 13 studies) (Akbari et al., 2022; Ardakanizade, 2018; Da Silva Jr et al., 2012; Esfahanni et al., 2014; Farhadi et al., 2024; Ghorbanzadeh et al., 2017; Iemitsu et al., 2006; Karisa, Sylviana, Goenawan, et al., 2025; Pourheydar et al., 2020; Rezaei et al., 2022; Sabzevari Rad et al., 2020; Soori et al., 2022; Sylviana et al., 2022), Sprague–Dawley rats (*n* = 5) (Hassan & Kamal, 2013; Ma et al., 2025; Marini et al., 2008; Verboven et al., 2019; Yeo & Lim, 2022), and C57BL/6 mice (Xiao et al., 2016), BALB/c mice (Sylviana et al., 2018), Swiss mice (Bellafiore et al., 2019), Zebra fish (Chen et al., 2021), and NMRI mice (Olamazadeh et al., 2025). Most studies used male animals (*n* = 20); only three studies included females (Da Silva Jr et al., 2012; Ma et al., 2025; Rezaei et al., 2022).

Across studies, angiogenesis regulators were assessed at miRNA, mRNA, and protein levels using PCR (*n* = 15), Western blot (*n* = 9), ELISA (*n* = 7), and immunofluorescence (*n* = 2). Structural or tissue‐level vascular assessment was performed using histology (e.g., H&E) and/or immunohistochemistry (IHC); however, eight studies did not report histological outcomes (Akbari et al., 2022; Ardakanizade, 2018; Esfahanni et al., 2014; Karisa, Sylviana, Goenawan, et al., 2025; Rezaei et al., 2022; Sabzevari Rad et al., 2020; Soori et al., 2022; Sylviana et al., 2022). All interventions were compared with a sedentary control group.

Based on the interventions provided to participants, the frequency, intensity, type, and duration of exercise varied across studies. The frequency of exercise ranged from 3 to 5 times per week, with a training period of 15 days to 12 weeks. The majority of the protocols ranged from 4 to 8 weeks, with each session lasting 30 to 240 min per day. The intensity varied widely, from moderate to high. However, in this study, it was difficult to assess the intensity of the exercise because the data were incomplete and did not include VO_2_ max or other markers of maximum exercise capacity. We focused on analyzing the differences in the types of exercise given. Of the 23 studies included, there were continuous training in the form of treadmill or running (*n* = 11) (Akbari et al., 2022; Bellafiore et al., 2019; Farhadi et al., 2024; Ghorbanzadeh et al., 2017; Karisa, Sylviana, Goenawan, et al., 2025; Marini et al., 2008; Pourheydar et al., 2020; Soori et al., 2022; Sylviana et al., 2022; Verboven et al., 2019; Yeo & Lim, 2022), swimming (*n* = 8) (Ardakanizade, 2018; Chen et al., 2021; Da Silva Jr et al., 2012; Hassan & Kamal, 2013; Iemitsu et al., 2006; Ma et al., 2025; Sylviana et al., 2018; Xiao et al., 2016), high intensity interval training (HIIT) (*n* = 5) (Akbari et al., 2022; Rezaei et al., 2022; Sabzevari Rad et al., 2020; Soori et al., 2022; Verboven et al., 2019), Resistance training or strength training (*n* = 3) (Esfahanni et al., 2014; Olamazadeh et al., 2025; Yeo & Lim, 2022), and combined training (*n* = 1) (Yeo & Lim, 2022).

### 3.3 Main findings

To summarize the evidence regarding exercise‐induced cardiac angiogenesis adaptation, all key outcomes from the included studies were extracted and standardized in Table 2. The analysis was conducted based on the type of exercise performed, which was then categorized into five themes: (1) Swimming‐based aerobic exercise, (2) continuous training (treadmill/running), (3) interval training/high intensity interval training (HIIT), (4) resistance training, and (5) combined training.

#### 3.3.1 Swimming training

In swimming training, the most consistent pattern of findings shows that hemodynamic and metabolic stimuli drive activation of the VEGF axis (VEGF along with increased Flt‐1/VEGFR1 and Flk‐1/VEGFR2), which then flows to PI3K/Akt and increases eNOS and p‐eNOS, thereby increasing NO bioavailability and leading to increased capillary density and myocyte capillary ratio (Iemitsu et al., 2006). In the post‐transcriptional process, the angiogenic response is also locked by miR‐126, which increases in parallel with the activation of Raf‐1/MAPK/ERK and PI3K/Akt/eNOS. At the same time, Spred‐1 (an inhibitor of the Ras/Raf pathway) decreases, thereby strengthening the pro‐angiogenic signal (Da Silva Jr et al., 2012). In the more chronic adaptation spectrum, several swimming studies show a shift from acute hypoxia to long‐term remodeling: PGC‐1α increases, accompanied by decreased HIF‐1α (Sylviana et al., 2018), as well as activation of matrix remodeling and vessel maturation modules (e.g., VEGF‐B/MEF2C/MMP‐2 and ANG‐1/HDAC4 dynamics) running parallel to increased NO/oxidative stress (Ardakanizade, 2018). Additional evidence of an upstream driver also emerges through the TESC‐C‐SRC/IGF1R‐PI3K/pAKT‐VEGFA/VEGFR2 pathway (Ma et al., 2025) and through vascular stabilization signaling involving CD34/PDGF, consistent with capillarization and physiological remodeling (Xiao et al., 2016).

#### 3.3.2 Continuous training (treadmill/running)

In continuous treadmill/running‐based training, the induction of angiogenesis is believed to be influenced by hypoxic conditions characterized by increased HIF‐1α along with VEGF and pro‐angiogenic receptors (e.g., KDR/VEGFR2) associated with endothelial sprouting (vWF+) and increased capillary index; when the stimulus is stopped (detraining), these signals decrease but some vascular adaptations persist (Marini et al., 2008). In continuous running, downstream activation is indicated by p‐Akt and p‐ERK1/2, accompanied by increases in miR‐210 (hypoxamiR) and miR‐126, as well as structural evidence of increased CD31+ (Ghorbanzadeh et al., 2017). On the other hand, treadmill data also show that the dominant pathway can shift with dose; for example, the initial response can be marked by HIF‐1α and changes in VEGFR‐1/Flt‐1, while increased workload strengthens VEGFR‐2/Flk‐1 and involves vasodilation/inflammatory mediators such as iNOS (Bellafiore et al., 2019). In an aging model, exercise increased VEGF–NF‐κB and the endothelial marker CD31, along with a decrease in TSP‐1 (an anti‐angiogenic factor), indicating that exercise may release the angiogenic brake in conditions that tend to be pro‐angiogenic (Pourheydar et al., 2020). Interestingly, variations in intensity show mechanistic divergence: at some intensities, angiogenesis is more closely associated with HIF‐1α–VEGF, whereas at others it is more closely associated with PGC‐1α/VEGF (Sylviana et al., 2022).

#### 3.3.3 High intensity interval training/HIIT

In interval training and HIIT, findings indicate a stronger angiogenic response than with moderate intensity, particularly in vascular indicators (number of vessels/capillaries) and endothelial activation. Structurally, HIIT increases the number of vessels more than MIT (Verboven et al., 2019). Molecularly, a recurring pattern is the dominance of miR‐126 (often highest during intervals), followed by increases in VEGF and Raf‐1/MAPK activation, while Spred‐1 decreases, thereby releasing inhibition of the Ras/Raf axis (Akbari et al., 2022; Sabzevari Rad et al., 2020). Endothelial activation biomarkers also strengthened: CD34 and KDR/VEGFR2 expression increased (HIIT > MIT), along with a decrease in CRP, suggesting that angiogenesis proceeds in tandem with improvement in the systemic inflammatory milieu (Rezaei et al., 2022). However, the effects are not singular: a comparison of HIIT versus continuous exercise shows differences in mediators (e.g., VEGF is more prominent in HIIT, while FGF‐2 is more prominent in continuous exercise), while both can reduce myocardial fibrosis (Soori et al., 2022).

#### 3.3.4 Resistance training

In RT, pro‐angiogenic signals often appear but are not always immediately converted into meaningful differences, especially when the duration of exercise is relatively short. For example, increases in NO, VEGF, and Flt‐1 can be seen but are not yet significant compared to controls; the interpretation that emerges is the need for a duration of >8 weeks so that VEGF expression is not only attached to the endothelium but produces a more pronounced angiogenic effect (Esfahanni et al., 2014). More substantial evidence indicates that RT can increase VEGF (mRNA and protein), and IHC examination results strongly support angiogenesis, and this response correlates with irisin, the proposed mechanism points to the AMPK/PGC‐1α/FNDC5 axis, which further induces irisin and VEGF as drivers of angiogenesis (Olamazadeh et al., 2025). In studies comparing modalities, RT tends to stand out for increasing PGC‐1α and FLK‐1/VEGFR2 receptor levels. In contrast, increases in VEGF/eNOS may be smaller than with AE, suggesting that in RT, the strengthening of receptor response capacity and metabolic signaling may be the dominant pathway when the hypoxia‐VEGF drive is not as high as in AE (Yeo & Lim, 2022).

#### 3.3.5 Combined training

In combined training (CT), several findings suggest a more comprehensive profile: a prominent increase in eNOS and CD31 (similar to AE). At the same time, PGC‐1α may be higher than in specific single modalities, and FLK‐1/VEGFR2 remains elevated (Yeo & Lim, 2022). Narratively, this combination theme can provide comprehensive stimuli, namely vascular‐shear stimuli (NO‐eNOS), metabolic‐mitochondrial stimuli (PGC‐1α), hypoxia control (FIH/PHD), and increased muscle mass and strength obtained through combined RT and AE. Together, these create the most conducive environment for stable physiological angiogenesis. This is evidenced by CD31 immunohistochemistry results showing increased angiogenesis and capillary density in the CT group, supported by increased Ang‐1 and Ang‐2 expression, which stabilize and mature blood vessels.

The synthesis in Table 2 shows that exercise mode not only determines the magnitude of the angiogenic response, but also selects the physiological mechanisms and molecular pathways recruited: continuous aerobic exercise (swimming/treadmill) tends to be dominated by flow stimuli and stable oxygen demand, thereby highlighting the VEGF–VEGFR and PI3K/Akt axes–eNOS/NO axis (with HIF‐1α involvement in specific contexts), while interval/HIIT with intensity surges and pulsatile shear more consistently enhances endothelial activation via miR‐126 and endothelial markers (e.g., CD34/KDR), while RT, which is dominated by pressure load, is more dependent on exposure thresholds and can be highlighted through the PGC‐1α/FNDC5/irisin–VEGF axis. In addition to exercise type, intensity and duration act as dose regulators that determine whether the response is transient or manifests as stable microvascular remodeling; for example, RT requires more prolonged exposure (often >8 weeks) for the initial response to develop into clear angiogenesis, whereas on a treadmill, variations in duration (±15–45 days) can shift the regulator from HIF‐1α/VEGFR‐1 towards VEGFR‐2 enhancement, so that intensity gradation can change the “main driver” (HIF‐1α vs. PGC‐1α) even though both still lead to VEGF.

## 4 DISCUSSION

**FIGURE 2:**
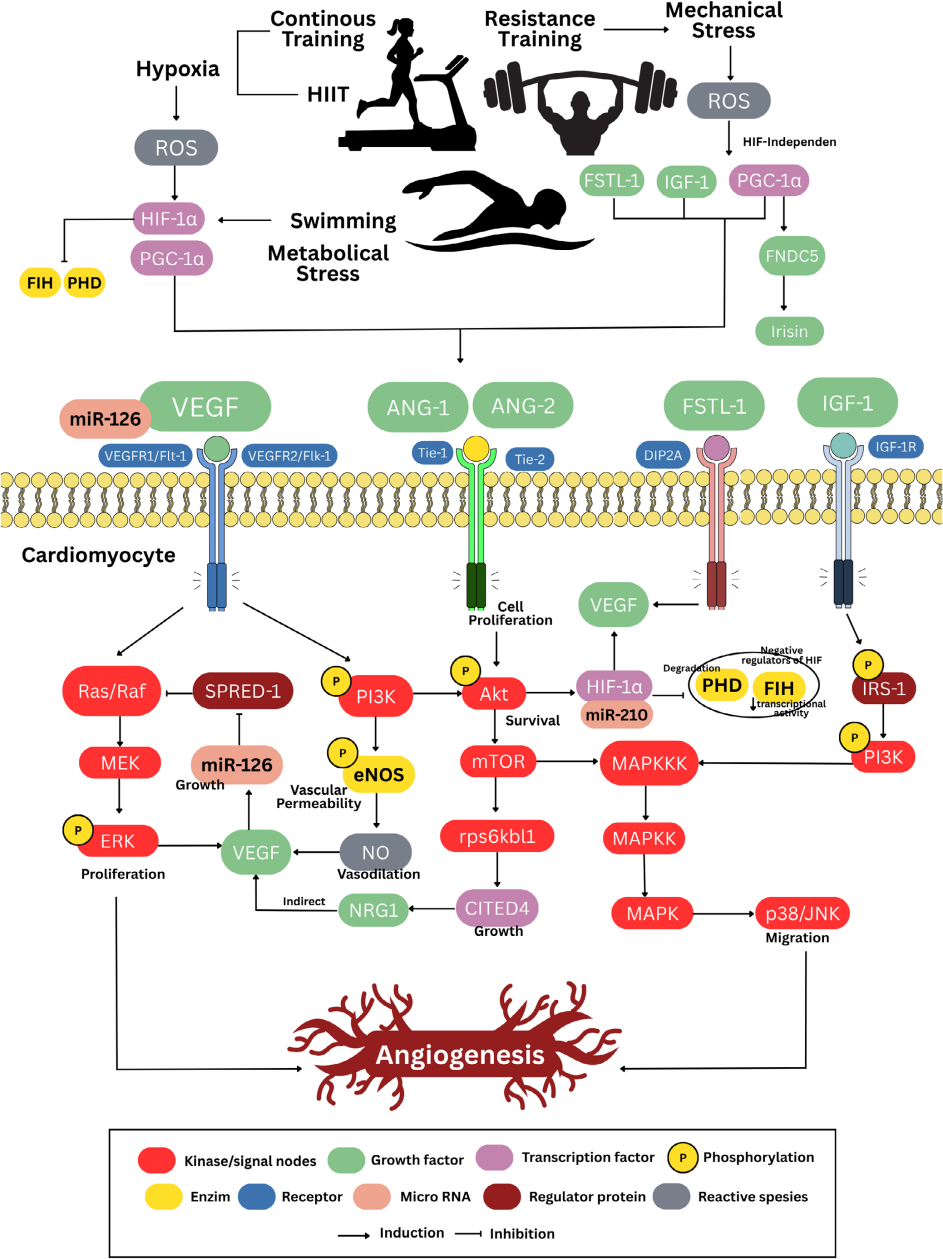
Proposed mechanism of the effects of different types of exercise on cardiac angiogenesis.

This scoping review synthesizes 23 studies comparing physiological targets elicited by different types of exercise to identify molecular mechanisms that can induce angiogenesis across various healthy populations. The main finding is that exercise in any modality induces cardiac angiogenesis, with distinct regulators depending on the physiological process, potentially reflecting the molecular physiology of angiogenesis being influenced by the type of exercise performed, even though the result is the same. A summary of the main findings of our analysis is presented in Table 2 and illustrated in Figure 2. Several important upstream regulators were identified, such as HIF‐1α, PGC‐1α, IGF‐1, Angiopoietin, FSTL‐1, and VEGF, which mediate the effects of exercise on cardiac angiogenesis.

Exercise is an effective non‐pharmacological therapeutic intervention for organ protection, as it promotes hypoxic adaptation (Brocherie, 2020). In this study, distinct angiogenic mechanisms were observed during different types of exercise. More specifically, during exercise, angiogenesis occurs through hypoxic mechanisms, which increase overall oxygen demand due to continuous activity. In RT, angiogenesis occurs due to mechanical stress on the heart wall, which triggers the release of several cardiokines. However, RT can cause local hypoxic and transient ischemic conditions, resulting from cardiac muscle hypertrophy. AE and RT also both increase ATP demand. This occurs because intense muscle contraction requires a large amount of energy to support muscle function. Increased ATP demand triggers increased mitochondrial activity and metabolism, which, in turn, indirectly elevates ROS production (Powers & Jackson, 2008). During RT, mechanical damage to the muscle triggers calcium release, which affects mitochondrial activity and ROS production. Although often considered harmful by‐products, ROS in the context of physical exercise play an important role as adaptive signals to enhance oxidative capacity and angiogenesis (Gomez‐Cabrera et al., 2015; Ji, 2015; Powers & Jackson, 2008).

### 4.1 Aerobic exercise with continuous training on a treadmill and swimming

AE performed on land and in water (swimming) has different physiological adaptations (Lu & Chang, 2012). The main properties of water that show clinical relevance are density, buoyancy, hydrostatic pressure, turbulence, viscosity, surface tension, and refractivity (Florian et al., 2013). In a swimming pool, individuals are influenced by these properties to keep their upper airways above the water surface, while on a treadmill, gravity and ground reaction forces are the influencing factors (Wilcock et al., 2006). The results of Moura et al. (2018) study show that sarcomere addition in cardiomyocytes is higher in rats trained to swim than in those trained on a treadmill. This is explained by the fact that hydrostatic pressure in water induces vasoconstriction, thereby increasing venous return and preload and promoting eccentric hypertrophy in accordance with Frank‐Starling's law (Fukuie et al., 2021). Increased vasoconstriction and stimulation of cardiomyocyte growth also increase post‐contraction load, thereby increasing heart wall thickness and producing stronger contractions (Duncker & Bache, 2008). Myocyte expansion increases oxygen and nutrient requirements to support normal heart function, a phenomenon known as physiological hypoxia (Shen et al., 2018). Hypoxia in the myocardium produces a series of metabolites that induce vasodilation and angiogenesis to maintain oxygen consumption (Auchampach, 2007). Myocyte injury due to continuous exercise contraction stimulates heart cells to release angiogenic factors and promote capillary expansion (Xiao et al., 2016). Thus, swimming exercise is considered a type of exercise that promotes angiogenesis more strongly than treadmill exercise, comparable to reactive hypertrophy (Yang et al., 2023).

Another factor that may contribute to increased cardiac angiogenesis is increased adrenergic activity. In vivo studies have shown that exercise increases shear stress and β3‐AR, which ultimately increases PI3K/Akt/eNOS activity, the main pathway for inducing cardiac angiogenesis (Farah et al., 2017; Suvorava & Cortese‐Krott, 2018). Both treadmill exercise and swimming have similar effects, but norepinephrine and adrenaline levels are higher than during continuous treadmill exercise (Baptista et al., 2008). This can be explained by the fact that during chronic exercise adaptation in the myocardium, there is humoral activation of the renin‐angiotensin system and the sympathetic nervous system, which release angiotensin II, norepinephrine, and endothelin‐1, which directly contribute to cardiac remodeling, hypertrophy, improved ventricular function, and stimulation of angiogenesis (Ramirez‐Hernandez et al., 2024). Studies in humans show different patterns of adaptation, with water‐based exercise tending to maintain myocardial oxygen balance and achieve higher stroke volume with better myocardial energy efficiency than land‐based exercise, even at the same inotropic mode (Fukuie et al., 2021). Although recent animal studies show that heart rate reduction, autonomic modulation, and ventricular remodeling are comparable between swimming and running (Lakin et al., 2018), miRNA participation differs between the two modes, suggesting distinct molecular mechanisms (Fernandes et al., 2015).

AEs such as running or swimming involve rhythmic contractions of large skeletal muscle masses over a natural period of time (e.g., 30–60 min), which are highly dependent on oxygen delivery to the active muscles to facilitate venous return and increase end‐diastolic volume (Fernandes et al., 2015). This type of exercise promotes cardiac angiogenesis by linking flow‐ and shear‐driven stimulation and metabolism to hypoxia‐sensitive transcription programs that increase pro‐angiogenic ligands. During sustained exercise, increased coronary flow and endothelial shear stress act as powerful upstream signals to promote vascularization (Yang et al., 2023). Increased myocardial oxygen demand can create a temporary local supply–demand imbalance, leading to increased ROS and stabilization of HIF‐1α (Bellafiore et al., 2019; Prior et al., 2004). Mechanistically, oxygen sensors regulate HIF‐1α availability (PHD promotes HIF‐1α degradation; FIH limits its transcriptional activity), such that decreased oxygen pressure/redox tone changes during exercise support HIF‐1α transcriptional output, including VEGF induction and hypoxia programs like miR‐210 (Bei et al., 2024; Bellafiore et al., 2019; Ghorbanzadeh et al., 2017; Iemitsu et al., 2006; Karisa, Sylviana, Goenawan, et al., 2025). In line with HIF‐dependent signaling, AE also increases PGC‐1α as a metabolic adaptation. PGC‐1α promotes VEGF via ERRα independently of HIF, providing a second pathway by which sustained aerobic activity can maintain an angiogenic environment even when hypoxia signaling is low (Leick et al., 2009; Sylviana et al., 2018).

At the effector level, increased VEGF activates its receptors on vascular endothelium. Functionally, VEGFR2/Flk‐1 is the dominant signaling receptor that activates the PI3K/Akt and Ras/Raf/MEK/ERK cascades (Bellafiore et al., 2019; Da Silva Jr et al., 2012; Pourheydar et al., 2020). Akt phosphorylation drives eNOS activation, increasing NO availability and supporting vasodilation, endothelial survival, and conditions conducive to bud formation. Meanwhile, ERK signaling supports the proliferative response. A key enhancer in this network is miR‐126, which amplifies angiogenic signaling by targeting negative regulators, such as SPRED‐1 (removing inhibition of MAPK output), and by supporting PI3K pathway tone. Experimentally, miR‐126 reduction blunts ERK/Akt phosphorylation and weakens VEGF‐related endothelial activation (Akbari et al., 2022; Da Silva Jr et al., 2012; Ghorbanzadeh et al., 2017; Sabzevari Rad et al., 2020). However, myocardial signaling of HIF‐1α, VEGFR‐1/Flt‐1, VEGFR‐2/Flk‐1, and iNOS/eNOS can shift across different exercise loads and time points, underscoring that aerobic angiogenesis is dynamically regulated through intensity and duration (Bellafiore et al., 2019; Hassan & Kamal, 2013; Karisa, Sylviana, Goenawan, et al., 2025; Sylviana et al., 2022; Verboven et al., 2019). This is supported by other studies showing that both swimming and treadmill running, whether of short or long duration, influence blood vessel adaptation in rats with myocardial ischemia (Budiono et al., 2021, 2025).

Ultimately, blood vessel formation and function depend not only on growth signals (VEGF) but also on maturation/stabilization pathways (Ardakanizade, 2018). The ANG‐1/ANG‐2‐Tie2 axis modulates endothelial survival and vascular stabilization (Ardakanizade, 2018; Yeo & Lim, 2022). ANG‐1 generally promotes Tie2/PI3K/Akt signaling and vascular quiescence, whereas ANG‐2, in the presence of VEGF signaling, can facilitate remodeling (Fagiani & Christofori, 2013; Thurston & Daly, 2012). This provides a mechanistic basis for incorporating ANG/Tie nodes into the AE model during the transition from initial endothelial activation to stable microvascular remodeling. In parallel, endothelial homeostasis is shaped by the balance between pro‐angiogenic and angiostatic mediators such as thrombospondin‐1 (TSP‐1), a potent endogenous inhibitor that counteracts VEGF activity and can suppress NO‐dependent endothelial responses (Isenberg et al., 2005; Pourheydar et al., 2020). In aging conditions, this balance tends to shift towards reduced capillarity and lower VEGF signaling, whereas physical exercise can partially restore VEGF pathway signaling in older myocardium (Chen et al., 2011; Zhang et al., 2023).

### 4.2 High intensity interval training (HIIT)

HIIT is considered more effective than moderate continuous training (MCT) because it combines higher glycolytic and oxidative metabolic demands (Abe et al., 2015; Milanović et al., 2015; Seeger et al., 2015). Mechanistically, the upstream trigger of angiogenesis in HIIT is not stable hypoxia, as in continuous exercise, but rather the repeated cycles of hypoxia‐reoxygenation and pulsatile shear stress induced by intensity surges (Soori et al., 2022). In the intense work phase, increased cardiac output and flow velocity sharply increase the antegrade shear rate, which then decreases during the recovery phase. This pattern triggers a burst of ROS that functions as a redox signal (including through NADPH oxidase activation) and enhances endothelial mechanical transduction and adaptation processes (Chatterjee, 2018; Rezaei et al., 2022). At the level of the oxygen sensor, HIF‐1α stabilization follows a general mechanism involving oxygen sensor inhibition and FIH modulation (Koyasu et al., 2018). However, in HIIT, hypoxia tends to be more transient and intermittent due to recovery. This is supported by data showing that muscle oxygenation (SMO_2_) decreases significantly and, in supramaximal protocols, can reach very deep deoxygenation. Consistent with the concept that at severe‐extreme intensities, O_2_ demand can exceed convective delivery capacity, thereby increasing the demand‐delivery mismatch and local deoxygenation (Paquette et al., 2019; Perentis et al., 2021; Yogev et al., 2024). The combination of intermittent hypoxia and pulsatile shear serves as a strong endothelial stimulus (Zhou et al., 2014) and has the potential to amplify HIF‐VEGF signaling, thereby enhancing the angiogenic response (Sabzevari Rad et al., 2020; Verboven et al., 2019).

Downstream, strong shear in HIIT activates the mechanosensory junctional complex (mis. PECAM‐1/VE‐cadherin/VEGFR2). It can activate VEGFR2 in a ligand‐independent manner, transmitting signals to PI3K/Akt/eNOS, thereby increasing NO bioavailability for vasodilation, survival, and endothelial sprouting (Hanson & Casey, 2023; Power et al., 2024). Consistently, Soori et al. (2022) reported greater increases in VEGF after HIIT than after continuous treadmill. This increase in VEGF expression in the myocardium correlates with increased cardiac tissue capillary density and increased PI3K/Akt/eNOS/NO pathways in the HIIT group. NO production has also been reported to be increased through the expression of IGF‐1 and FGF‐2 (Moran et al., 2007). In HIIT training, although other studies show that FGF‐2 and IGF‐1 can increase to a similar extent, with the effects of MCT sometimes being more pronounced (Soori et al., 2022). These findings confirm that the difference in HIIT primarily lies in the stimulus that enhances endothelial activation and the VEGF axis, rather than merely in the presence of growth factors.

Functionally, extensive evidence shows that physical exercise that stimulates hypoxia, including HIIT, increases angiogenesis, improves endothelial function, and suppresses myocardial fibrosis regardless of the type of exercise (Kwak et al., 2011; Shave & Oxborough, 2016; Yang et al., 2011). The uniqueness of HIIT is also evident in structural remodeling, where AWT and PWT, as well as markers of left ventricular wall hypertrophy, tend to increase more than MCT (Cassidy et al., 2016; Verboven et al., 2019). In this context, the relationship between angiogenesis and hypertrophy has been emphasized previously, with physiological hypertrophy always accompanied by angiogenesis, as demonstrated by paracrine effects and the secretion of NO and growth factors (e.g., VEGF and NRG‐1) (Walsh & Shiojima, 2007). In line with this, Verboven et al. (2019) reported that only HIIT increases capillary density. Adequate capillarity facilitates greater oxygen delivery to the heart muscle and supports myocardial metabolism (Park et al., 2016). This is crucial because oxygen consumption during HIIT approaches maximum levels and ATP production increases, so vascularization is needed to meet this demand (Kolwicz, 2018). At the post‐transcriptional level, HIIT also increases the expression of regulators such as miR‐126, which suppresses VEGF pathway inhibitors (SPRED‐1 and PIK3R2), thereby strengthening Ras/MAPK and PI3K/Akt/eNOS and increasing VEGF (Da Silva Jr et al., 2012; Ghorbanzadeh et al., 2017; Jansen et al., 2013). Other findings indicate that HIIT increases miR‐126 and VEGF via Raf‐1 activation and Spred‐1 (Sabzevari Rad et al., 2020). Other miR profiles (miR‐222/miR‐29a) have also been reported to be more beneficial in HIIT than in MCT, and this effect may be further enhanced by metabolic improvements, such as increased GLUT4 and insulin sensitivity (Akbari et al., 2022; Jansen et al., 2013).

### 4.3 Resistance training (RT)

Resistance or strength training, such as weight lifting, involves smaller muscle mass. However, strength contraction increases with repeated repetitions until fatigue and increases systemic vascular resistance (excess pressure load or increased heavier isometric contraction) (Fernandes et al., 2015). Unlike running on a treadmill or swimming, RT triggers cardiac angiogenesis through pressure load/afterload and repeated mechanical stress on the myocardium, thereby activating mechanotransduction‐redox signaling and increasing the release of pro‐angiogenic factors from cardiomyocytes (Karisa, Sylviana, Rosdianto, et al., 2025; Xi et al., 2021). Experimentally, cyclic mechanical stretch in adult cardiomyocytes can increase VEGF secretion several‐fold, and this increase correlates with NF‐κB activation. Therefore, it makes sense that RT (with strong contractions and increased wall stress) increases the pro‐angiogenic secretome, which then acts paracrinally on the coronary endothelium (Leychenko et al., 2011). Interestingly, HIF‐1α need not be viewed solely as a hypoxia‐dependent pathway; evidence indicates that mechanical stress can induce HIF‐1α protein and increase VEGF expression in isolated heart preparations (Kim et al., 2002). Thus, in RT, the HIF‐1α/miR‐210 pathway can still induce VEGF but runs parallel to the more dominant mechanical‐growth pathway, which is achieved through continuous load increase and repetition (Karisa, Sylviana, Rosdianto, et al., 2025).

The key distinguishing feature of RT compared to continuous aerobic exercise/HIIT is that RT more strongly locks the IGF‐1/PI3K/Akt growth‐coupling axis, which coordinates physiological myocardial growth with microvascular expansion (Al‐Sarraf & Mouihate, 2022; Cheng et al., 2025). Strong causal evidence is described in the myocardial Akt activation model. Initially, Akt enhances coronary angiogenesis by inducing VEGF and Ang‐2 via mTOR (Shiojima & Walsh, 2006). This provides a biological basis for why the downstream mTOR/RPS6KB1 node is crucial in enhancing protein synthesis capacity, enabling the adaptation program to proceed. At the same time, its angiogenic output emerges when myocardium‐produced VEGF/Ang‐2 activates endothelial cells for proliferation, sprouting, and maturation (Cheng et al., 2025). In this case, the angiogenic response to this type of exercise is also accompanied by physiological hypertrophy, balancing growth and vascularization so that it does not shift into dysfunction (Oka et al., 2014). At the growth program level, RT can interact with the C/EBPβ–CITED4 module and miR‐222, thereby enabling RT angiogenesis to be conceptualized as growth‐matched angiogenesis (Barauna et al., 2007; Cheng et al., 2025).

In addition to the IGF‐1/Akt/mTOR axis, RT often stands out through metabolic myokine/cardiokine enhancers, distinguishing it from pure aerobic metabolism. PGC‐1α is one of the HIF‐independent pathways that can induce VEGF expression through ERRα coactivation, allowing RT to increase VEGF even when hypoxia is not the primary trigger (Arany et al., 2008; Rowe et al., 2012). PGC‐1α activation can lead to FNDC5, another form of Irisin. Irisin has been shown to improve endothelial function through increased AMPK/Akt/eNOS phosphorylation and NO production (Olamazadeh et al., 2025). FSTL‐1, as a myokine/cardiokine, is also known to induce angiogenesis through RT (Karisa, Sylviana, Fitria, & Setiawan, 2025; Xi et al., 2021). FSTL‐1 binds DIP2A, activating Smad2/3 and increasing endothelial proliferation and VEGF‐A activation in rats with myocardial infarction (Li et al., 2023). FSTL‐1 from skeletal muscle also induced cardiac angiogenesis through the DIP2A‐Smad2/3 pathway (Xi et al., 2021). Consistent with recent studies, RT in healthy experimental animals increased pure cardiac FSTL‐1, in line with increased VEGF (Karisa, Sylviana, Rosdianto, et al., 2025). With stimuli driven by pressure load and growth factor coupling and reinforced by myokines/cardiokines, the resulting angiogenesis mechanism is not merely a response to hypoxia but rather microvascular remodeling coupled with physiological growth. However, it requires a high exposure threshold in terms of duration and intensity to induce stable, significant angiogenesis (Leychenko et al., 2011). The study by Karisa, Sylviana, Rosdianto, et al. (2025) demonstrated that chronic RT can induce a more stable VEGF response than acute exercise. This is supported by Padilha et al., which found that moderate‐ and high‐intensity RT can induce skeletal muscle hypertrophy, whereas low‐intensity RT has no effect (Padilha et al., 2019).

### 4.4 Combined training (CT)

CT combines two main stimuli: shear stress from the aerobic component and mechanical load from the resistance component. Several studies have shown that AE alone produces better results in cardiorespiratory fitness than CT or RT (Alemayehu & Teferi, 2023). We hypothesize that this is related to the heart's adaptation mechanisms to different forms of exercise, including angiogenesis. In AE alone, the dominance of stable flow/oxygen often more clearly increases HIF‐1α/VEGF. In contrast, RT or CT often show increased VEGFR2/Flk‐1, reflecting increased endothelial sensitivity/proliferation when the VEGF stimulus is not at its highest. This means that CT acts as a stabilizing effort, while AE provides a strong initial stimulus (Bellafiore et al., 2019; Yeo & Lim, 2022). In the study by Yeo and Lim (2022), eNOS increased in AE and CT but was not prominent in RT, a pattern that can be explained by shear‐dependent signaling. This may be due to no change in plasma NOX and eNOS levels, despite increased endothelin‐1 in RE, which inhibits eNOS activity (Cocks et al., 2014; Hasegawa et al., 2018). In addition, Flk‐1 also appeared to increase significantly in the RT and CT groups but not in the AE group, although it was higher than the control group, and vice versa for VEGF (Yeo & Lim, 2022). Flk‐1 can compensate for VEGF deficiency, which may be caused by the intensity and duration of exercise (Bellafiore et al., 2019). Interestingly, HIF‐1α increased significantly only in AE, whereas PGC‐1α/ANG‐1/ANG‐2 increased proportionally across all exercise types compared to the control (Yeo & Lim, 2022). This indicates the specificity of the exercise types: AE induces angiogenesis via hypoxia signaling and mitochondrial metabolism, whereas RT induces angiogenesis via a pathway independent of HIF (Karisa, Sylviana, Rosdianto, et al., 2025). Unfortunately, we found only one study that examined the effects of CT on cardiac angiogenesis, particularly from a molecular perspective. Therefore, this study warrants further exploration.

The local hypoxic effect of AE, combined with mechanical stretching that strengthens heart muscle via RT, creates a stronger environment for angiogenic signaling, leading to vascular regeneration and improved cardiac function (Saati‐Zarei et al., 2023; Yeo & Lim, 2022). Although AE has been shown to provide better results than RT, loss of muscle mass and strength is associated with a decreased ability of the heart to regenerate, especially in the aged (Alves et al., 2020; Volaklis et al., 2013; Yeo & Lim, 2022). In hypertensive populations, it was found that CT can provide comparable results to AE alone if the aerobic stimulus portion is increased (Alemayehu & Teferi, 2023). However, other studies have noted that CT has a stronger association with mortality from coronary heart disease (CHD) and other diseases compared to AE or RT alone (Paluch et al., 2024). This is because CT provides broader complementary adaptations (increased oxidative capacity and muscle strength), making it more relevant for long‐term clinical outcomes (Alemayehu & Teferi, 2023; Paluch et al., 2024; Tryfonos et al., 2021).

## 5 STRENGTHS AND LIMITATIONS OF LITERATURE

This study's main strength is its focus on evidence that is truly cardiac‐specific (biomarkers from the myocardial/coronary network and/or microvascular structural indicators). The scoping review methodology is relatively transparent, and the synthesis of findings does not stop at the magnitude of the effect, but also addresses the mode of exercise with the recruited physiological‐molecular pathways (e.g., VEGF‐VEGFR, PI3K/Akt‐eNOS/NO, HIF‐1α, PGC‐1α, and miRNA). However, limitations include protocol heterogeneity and incomplete reporting of intensity, limiting dose–response analysis, and an imbalance in the evidence for RT and, especially, CT, leading to weak mechanistic inferences for both modes. Furthermore, some studies lacked histological verification and showed gender bias, as the majority used male subjects, limiting generalizability to females.

## 6 CONCLUSION

Based on a mapping of 23 studies on physiological animal models, this paper concludes that various types of exercise can induce cardiac angiogenesis. However, the mode of exercise determines which pathway is recruited. Thus, the type of exercise acts as a mechanistic determinant rather than merely a variation in stimulus dose. In general, continuous aerobic training and HIIT tend to emphasize the VEGF/VEGFR and PI3K/Akt‐eNOS/NO axes, with greater involvement of HIF‐1α. Meanwhile, other modes, such as RT, show different regulatory patterns, leaning more towards HIF‐independent angiogenesis. As for CT, it has a mechanism that is more dominant towards the aerobic pathway at higher aerobic intensities, and the effects of RT combined with AE exert greater influence on muscle strengthening and a more comprehensive increase in metabolism. Given the limitations of the evidence, particularly for RT and CT, and the dominance of male population studies, future research could explore the mechanisms of RT and CT on cardiac angiogenesis and involve female animals to examine mechanistic differences between exercise modes and to strengthen FITT reporting for safer translation.

## AUTHOR CONTRIBUTIONS

PK, NS, and SS: Conceptualization and methodology; PK, SS, and NS: Software; NS, SS, AMR, and HG: Validation; PK, NS, SS, and HG: Formal analysis; NS, SS, MRAAS, and AMR: Investigation; PK, NS, SS, MRAAS, and AMR: Resources; PK, NS, MRAAS, and AMR: Data curation; PK and NS: Writing–original draft preparation; SS, MRAAS, AMR, and HG: Writing–review and editing; PK and MRAAS: Visualization; NS, SS, MRAAS, AMR, and HG: Supervision. All authors have read and agreed to the published version of the manuscript.

## FUNDING INFORMATION

This research received no external funding.

## CONFLICT OF INTEREST STATEMENT

The authors declare no competing interests.

## Supporting information


Tables S1–S2.

## Data Availability

All data generated or analyzed during this study are included in Tables S1 and S2.
