# Supplementary material for: Different types of exercise and myocardial angiogenesis regulation: A scoping review of cardiac‐specific evidence in animal models
Source: Physiol Rep. 2026 Mar 4;14(5):e70775. doi: 10.14814/phy2.70775 (PMC12960063; doi:10.14814/phy2.70775)
Supplement: Supplementary file 1 — Tables S1–S2. [file PHY2-14-e70775-s001.docx]

Table S1. PRISMA-ScR checklist

| **SECTION** | **ITEM** | | **PRISMA-ScR** **CHECKLIST** **ITEM** | **REPORTED** **ON** **PAGE** **#** |
| --- | --- | --- | --- | --- |
| **TITLE** | | | | |
| Title | 1 | | Identify the report as a scoping review. | 1 |
| **ABSTRACT** | | | | |
| Structured summary | 2 | | Provide a structured summary that includes (as applicable): background, objectives, eligibility criteria, sources of evidence, charting methods, results, and conclusions that relate to the review questions and objectives. | 23-41 |
| **INTRODUCTION** | | | | |
| Rationale | 3 | | Describe the rationale for the review in the context of what is already known. Explain why the review questions/objectives lend themselves to a scoping review approach. | 70-124 |
| Objectives | 4 | | Provide an explicit statement of the questions and objectives being addressed with reference to their key elements (e.g., population or participants, concepts, and context) or other relevant key elements used to conceptualize the review questions and/or objectives. | 125-129 |
| **METHODS** | | | | |
| Protocol and registration | 5 | | Indicate whether a review protocol exists; state if and where it can be accessed (e.g., a Web address); and if available,  provide registration information, including the registration number. | N/A for protocol |
| Eligibility criteria | 6 | | Specify characteristics of the sources of evidence used as eligibility criteria (e.g., years considered, language, and publication status), and provide a rationale. | 161 |
| Information sources* | 7 | | Describe all information sources in the search (e.g., databases with dates of coverage and contact with authors to identify additional sources), as well as the date the most recent search was executed. | 142 |
| Search | 8 | | Present the full electronic search strategy for at least 1 database, including any limits used, such that it could be  repeated. | Supplementary File 2 (Table S2) |
| Selection of sources of  evidence† | 9 | | State the process for selecting sources of evidence (i.e., screening and eligibility) included in the scoping review. | 152 |
| Data charting process‡ | 10 | | Describe the methods of charting data from the included sources of evidence (e.g., calibrated forms or forms that have been tested by the team before their use, and whether data charting was done independently or in duplicate) and any processes for obtaining and confirming data from investigators. | 153-160 |
| Data items | 11 | | List and define all variables for which data were sought and  any assumptions and simplifications made. | 205-211 |
| Critical appraisal of individual sources of evidences | 12 | | If done, provide a rationale for conducting a critical appraisal of included sources of evidence; describe the methods used and how this information was used in any data synthesis (if appropriate). | N/A for protocol |
| Summary measure | 13 | | Not applicable for scoping review | N/A for protocol |
| Synthesis of results | 14 | | Describe the methods of handling and summarizing the data that  were charted. | 209-211 |
| Risk of bias across studies | 15 | | Not applicable for scoping review | N/A for protocol |
| Additional analyses | 16 | | Not applicable for scoping review | N/A for protocol |
| **RESULTS** | | | | |
| Selection of sources of evidence | 17 | Give numbers of sources of evidence screened, assessed for eligibility, and included in the review, with reasons for exclusions at each stage, ideally using a flow diagram. | | 214-221 |
| Characteristics of sources of evidence | 18 | For each source of evidence, present characteristics for which data were charted and provide the citations. | | 227-274 |
| Critical appraisal  within sources of evidence | 19 | If done, present data on critical appraisal of included sources of evidence (see item 12). | | N/A for protocol |
| Results of individual sources of evidence | 20 | For each included source of evidence, present the relevant data  that were charted that relate to the review questions and objectives. | | 278-292 |
| Synthesis of results | 21 | Summarize and/or present the charting results as they relate  to the review questions and objectives. | | 293-385 |
| Risk of bias across studies | 22 | Not applicable for scoping review | | N/A for protocol |
| Additional analyses | 23 | Not applicable for scoping review | | N/A for protocol |
| **DISCUSSION** | | | | |
| Summary of evidence | 24 | Summarize the main results (including an overview of concepts, themes, and types of evidence available), link to the review questions and objectives, and consider the relevance to key  groups. | | 387-660 |
| Limitations | 25 | Discuss the limitations of the scoping review process. | | 661 |
| Conclusions | 26 | Provide a general interpretation of the results with respect to the review questions and objectives, as well as potential implications and/or next steps. | | 673 |
| **FUNDING** | | | | |
| Funding | 27 | Describe sources of funding for the included sources of evidence, as well as sources of funding for the scoping  review. Describe the role of the funders of the scoping review. | | 704 |

* Where *sources* *of* *evidence* (see second footnote) are compiled from, such as bibliographic databases, social media platforms, and Web sites.

† A more inclusive/heterogeneous term used to account for the different types of evidence or data sources (e.g., quantitative and/or qualitative research, expert opinion, and policy documents) that may be eligible in a scoping review as opposed to only studies. This is not to be confused with *information* *sources* (see first footnote).

‡ The frameworks by Arksey and O’Malley and Levac and colleagues to the process of data extraction in a scoping review as data charting*.*

§ The process of systematically examining research evidence to assess its validity, results, and relevance before using it to inform a decision. This term is used for items 12 and 19 instead of "risk of bias" (which is more applicable to systematic reviews of interventions) to include and acknowledge the various sources of evidence that may be used in a scoping review (e.g., quantitative and/or qualitative research, expert opinion, and policy document).

*From:* Tricco AC, Lillie E, Zarin W, O'Brien KK, Colquhoun H, Levac D, et al. PRISMA Extension for Scoping Reviews (PRISMA- ScR): Checklist and Explanation. Ann Intern Med;169:467–473. doi: 10.7326/M18-0850

Table S2. Search Strategy

| **Databases** | **Search Strategy** | **Result** |
| --- | --- | --- |
| PubMed (MEDLINE) | #1  ((((((((((((((((((((((((((((((exercise[MeSH Terms]) OR (exercise therapy[MeSH Terms])) OR (animal physical conditioning[MeSH Terms])) OR (aerobic exercise[MeSH Terms])) OR (resistance training[MeSH Terms])) OR (endurance training[MeSH Terms])) OR (high intensity interval training[MeSH Terms])) OR (exercise[Title/Abstract])) OR (exercise training[Title/Abstract])) OR (training[Title/Abstract])) OR (physical activity[Title/Abstract])) OR (physical exercise[Title/Abstract])) OR (physical fitness[Title/Abstract])) OR (aerobic[Title/Abstract])) OR (endurance[Title/Abstract])) OR (endurance training[Title/Abstract])) OR (interval training[Title/Abstract])) OR (HIIT[Title/Abstract])) OR (high intensity interval[Title/Abstract])) OR (sprint[Title/Abstract])) OR (moderate intensity continous[Title/Abstract])) OR (MICT[Title/Abstract])) OR (resistance training[Title/Abstract])) OR (strength training[Title/Abstract])) OR (weight training[Title/Abstract])) OR (circuit training[Title/Abstract])) OR (swimming[Title/Abstract])) OR (treadmill[Title/Abstract])) OR (concurrent training[Title/Abstract])) OR (combined training[Title/Abstract])) OR (combined exercise[Title/Abstract]) | 1,350,375 |
|  | #2  (angiogenesis[Title/Abstract])) OR (neovascular*[Title/Abstract])) OR (arteriogenesis[Title/Abstract])) OR (capillar*[Title/Abstract])) OR (capillary density[Title/Abstract])) OR (microvessel density[Title/Abstract])) OR (vascular remodeling[Title/Abstract])) OR (VEGF[Title/Abstract])) OR (vascular endothelial growth factor[Title/Abstract])) OR (VEGFR*[Title/Abstract])) OR (Flk-1[Title/Abstract])) OR (Flt-1[Title/Abstract])) OR (HIF-1[Title/Abstract])) OR (eNOS[Title/Abstract])) OR (nitric oxide synthase[Title/Abstract])) OR (angiopoietin[Title/Abstract])) OR (Tie2[Title/Abstract])) OR (FGF*[Title/Abstract])) OR (fibroblast growth factor[Title/Abstract])) OR (PGC-1[Title/Abstract])) OR (PPARGC1A[Title/Abstract])) OR (FSTL1[Title/Abstract])) OR (CD31[Title/Abstract])) OR (PECAM1[Title/Abstract])) OR (CD34[Title/Abstract])) OR (yWF[Title/Abstract]) | 587,205 |
|  | #3  ((((((((heart[MeSH Terms]) OR (myocardium[MeSH Terms])) OR (coronary vessel[MeSH Terms])) OR (heart[Title/Abstract])) OR (cardiac[Title/Abstract])) OR (myocard[Title/Abstract])) OR (coronary[Title/Abstract])) OR (cardiac muscle[Title/Abstract])) OR (left ventricle[Title/Abstract]) | 1,350,375 |
|  | #1 AND #2 AND #3 | 1,467 |
| Scopus | #1  exercise OR "exercise training" OR "endurance training" OR "resistance training" OR "strength training" OR "weight training" OR HIIT OR "high intensity interval" OR "high-intensity interval" OR "aerobic exercise" OR treadmill OR sprint OR “moderate intensity continuous” OR swimming OR running OR cycling OR "wheel running" OR "ladder climbing" OR "combined training" OR “combined exercise” | 1,721,899 |
|  | #2  angiogenesis OR "angiogenic factor" OR neovascularization OR microvascular OR "vascular growth" OR "HIF 1" OR VEGF OR "Vascular endothelial growth factor" OR eNOS OR NOS3 OR "Nitric oxide" OR angiopoietin OR "angiopoietin 1" OR "angiopoietin 2" OR Tie2 OR TEK OR PDGF OR Notch OR DLL4 OR Jagged OR YAP OR TAZ OR microRNA OR miRNA OR exerkine OR irisin OR FNDC5 OR FSTL1 OR CD31 OR CD34 OR PECAM1 OR yWF OR PGC-1 OR FGF | 1,239,141 |
|  | #3  Heart OR myocardium OR “coronary vessel” OR cardiac OR myocard OR coronary OR “cardiac muscle” OR “left ventricle” | 283,598 |
|  | #1 AND #2 AND #3 | 1,620 |
| Springer Link | #1  exercise OR "exercise training" OR "endurance training" OR "resistance training" OR "strength training" OR "weight training" OR HIIT OR "high intensity interval" OR "high-intensity interval" OR "aerobic exercise" OR treadmill OR sprint OR “moderate intensity continuous” OR swimming OR running OR cycling OR "wheel running" OR "ladder climbing" OR "combined training" OR “combined exercise” | 532,411 |
|  | #2  angiogenesis OR "angiogenic factor" OR neovascularization OR microvascular OR "vascular growth" OR "HIF 1" OR VEGF OR "Vascular endothelial growth factor" OR eNOS OR NOS3 OR "Nitric oxide" OR angiopoietin OR "angiopoietin 1" OR "angiopoietin 2" OR Tie2 OR TEK OR PDGF OR Notch OR DLL4 OR Jagged OR YAP OR TAZ OR microRNA OR miRNA OR exerkine OR irisin OR FNDC5 OR FSTL1 OR CD31 OR CD34 OR PECAM1 OR yWF OR PGC-1 OR FGF | 929,473 |
|  | #3  Heart OR myocardium OR “coronary vessel” OR cardiac OR myocard OR coronary OR “cardiac muscle” OR “left ventricle” | 167,532 |
|  | #1 AND #2 AND #3 | 1,587 |
